# Reducing the Forensic Footprint with Android Accessibility Attacks

**DOI:** 10.1007/978-3-030-59817-4_2

**Published:** 2020-08-24

**Authors:** Yonas Leguesse, Mark Vella, Christian Colombo, Julio Hernandez-Castro

**Affiliations:** 8 Royal Holloway, University of London, Egham, UK; 9 National Research Council, Pisa, Italy; 10 Department of Computer Science, University of Malta, Msida, Malta; 11 School of Computing, Cornwallis South, University of Kent, Canterbury, UK

**Keywords:** Android security, Android accessibility attacks, Anti-forensics, Malware detection

## Abstract

Android accessibility features include a robust set of tools allowing developers to create apps for assisting people with disabilities. Unfortunately, this useful set of tools can also be abused and turned into an attack vector, providing malware with the ability to interact and read content from third-party apps.

In this work, we are the first to study the impact that the stealthy exploitation of Android accessibility services can have on significantly reducing the forensic footprint of malware attacks, thus hindering both live and post-incident forensic investigations. We show that through Living off the Land (LotL) tactics, or by offering a malware-only substitute for attacks typically requiring more elaborate schemes, accessibility-based malware can be rendered virtually undetectable.

In the LotL approach, we demonstrate accessibility-enabled SMS and command and control (C2) capabilities. As for the latter, we show a complete cryptocurrency wallet theft, whereby the accessibility trojan can hijack the entire withdrawal process of a widely used app, including two-factor authentication (2FA). In both cases, we demonstrate how the attacks result in significantly diminished forensic evidence when compared to similar attacks not employing accessibility tools, even to the extent of maintaining device take-over without requiring malware persistence.

## Introduction

Mobile devices have evolved significantly, both in sophistication and market adoption, over the last decade. In 2019, Google announced that there were over 2.5 billion active Android devices [1], turning it into the largest operating system in terms of existing users. From a cybercriminal’s perspective, this constitutes a large and attractive target. Indeed, the increase in mobile device usage has resulted in a steady rise in mobile malware over recent years [2].

Moreover, financial services such as banking and cryptocurrency exchanges are firmly moving towards mobile platforms, turning Android devices into an appealing and potentially very profitable target. Malware detection on Android devices follows a typical multi-stage approach, aiming for early detection and removal. Google relies on automated malware analysis [3] to scan all apps uploaded to their app store. This is complemented by on-device scans, where additional information related to the actual operational environment becomes available. Besides this, the user is continuously prompted by applications requesting sensitive permissions or engaging in potentially dangerous operations [4], both aimed at quickly detecting, exposing and stopping app misdemeanour.

However, at times stealthy malware does still make it through all these protective layers, ultimately getting exposed during later infection stages through indicators of compromise such as expensive mobile service bills or a severely reduced battery life. At this point, and incident response investigation will try to trace attack activity back to the enabler malware artefacts [5] and take the necessary rectification steps. While current malware already uses a combination of emulation detection [6], code obfuscation and social engineering tricks [7] for evading detection, we argue that the addition of lesser-known accessibility services can significantly hamper malware detection and investigation.

The abuse of accessibility services can result in a significant reduction in the number of malware-specific components, which can, in some cases, not be necessary at all even while the device remains under full control of the attacker. The net result is that the number of forensic artefacts or the overall forensic footprint, heavily relied upon by on-device malware detectors and incident response tools is vastly reduced. It is also possible to not only diminish but also to manipulate this footprint so that the artefacts left become misleading, so that the true origins, causes and perpetrators of the attack remaining elusive.

Typical sources of forensic artefacts include the suspicious binary itself, probes and logs generated during its execution, and memory artefacts left during and after execution [8], to mention only a few. For example, when looking for an SMS-sending malware, a detection/response tool may look for the SMS-sending code within the app’s decompiled resources, as well as for the artefacts left after the execution of the SMS-sending functionality. Android performs in the background a sequence of events that allow for the SMS sending to take place. These include data repository insertions, inter-app communications, and radio communications. Remnants from all these activities could expose the malware and therefore constitute valuable forensic artefacts. Any malware that attempts to reduce this forensic footprint will, at the same time, benefit from a reduced chance of detection.

In this work, we demonstrate that the deliberate misuse of the Android accessibility service offers one such possible method of evasion. While initially conceived to render all Android apps automatically accessible to all end-users, this feature ended up being abused by app developers for all sorts of inter-app communication [9], e.g. password managers. Eventually, malware authors aiming to bypass Android’s security model also caught up with this feature. Since all previous efforts to remove re-purposed accessibility apps or to render accessibility safer have proven unsuccessful, accessibility has now become the Achilles heel [10] of Android’s security. The misuse of accessibility services is increasingly becoming a staple of Android malware, ranging from banking trojans, e.g. Gustuff [11] and EventBot [12], to fully-fledged malware bots, e.g. Cerberus [11] and DEFENSOR ID [10].

While previous work primarily focuses on the dangers of accessibility in terms of malicious capabilities, in this paper we study for the first time the increased stealth features of this attack strategy. We show that through Living off the Land (LotL) tactics, which is already very popular in Windows malware [13], accessibility malware can be rendered virtually undetectable, even by state-of-the-art detection and recovery tools.

Our study confirms that accessibility attacks can significantly reduce the forensic footprint when compared to more standard, non-accessibility attacks. Specifically, we make use of a proof-of-concept malware that abuses SMS functionality and breaks the two-factor authentication (2FA) of a widely used cryptowallet app. We are able to demonstrate a significantly reduced forensic footprint in the sources of evidence used by current malware detection and incident response tools. In summary, we make the following contributions:

- We demonstrate that through the abuse of the accessibility service, LotL tactics can be performed on Android (Subsects. 3.1–3.4).
- We then describe a novel way to implement a cryptocurrency wallet theft. The accessibility malware hijacks the entire withdrawal process, including 2FA authentication, thus providing a malware-only alternative to more elaborate cybercrime schemes.
- This averts forensic evidence on other channels (Subsect. 3.5). Our research confirms that accessibility attacks significantly reduce the forensic footprint when compared to non-accessibility attacks (Sect. 4).

## Background

### Android Attack Vectors

Android malware typically consists of packed or embedded malicious code inside seemingly or truly benign applications. The malicious code then performs malign operations on the victim’s device by executing a series of commands either hard-coded or received through the command and control (C2) channel [14]. However, if this code requires the use of OS features deemed as dangerous, it must request the appropriate permission or functionality. Examples of these are the SEND_SMS permission, or access to the camera. At this moment, malware must somehow trick the victim into enabling the relevant permission.

A more advanced vector, known as capability leak, involves abusing privileged components in third-party apps that do not adequately restrict access to their functionality. It is usually made possible through non-secured inter-app component communication channels [15]. It can result in a reduced forensic footprint since malware can perform the malign operation without explicitly requesting the associated permission or feature. However, the ability to carry out this capability leak attack is not very dependable as it usually relies on insecure coding on the part of the third-party app developer and requires the vulnerable app to be installed on the device at the point of malware execution.

One particularly sought-after attack vector comprises vulnerabilities inside system code, whose exploitation result in device rooting [16]. However, while extremely powerful, in terms of stealth, this approach can be very noisy.

### The Accessibility Attack Vector

Android accessibility features are made available to any app requesting the BIND_ACCESSIBILITY_SERVICE permission. By simply sub-classing AccessibilityService, along with component registration, the app gains access to all GUI events of interest for any app. Its corresponding event handling code receives an AccessibilityEvent through which the GUI of a third-party app is abstracted as a tree of GUI elements along with all displayed information (possibly including credentials or other sensitive information) which are not protected by the importantForAccessibility mask. Apart from reading associated data, through the AccessibilityNodeInfo class, the accessibility service, in turn, can invoke further actions on the target app. While rendering every app on an Android device accessible to alternative means of interaction, accessibility services also introduced the nasty side-effect of potentially backdooring Android’s security model.

**Fig. 1.**
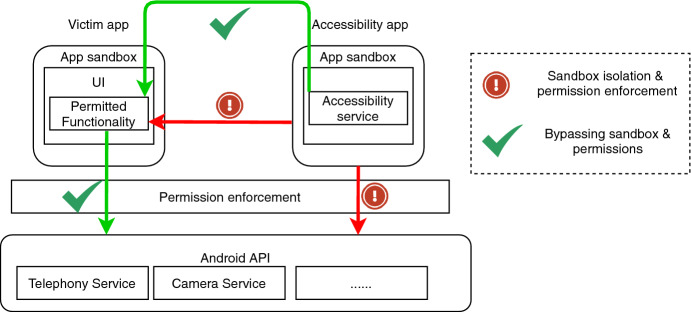
The accessibility attack vector bypasses Android’s permission and isolation-based security model.

This model is based on the principles of consent, isolation and containment [17] and it is surprising that it can be bypassed with a single abused permission [18] (Fig. 1). When compared to typical capability leaks, accessibility attacks are way more practical, from an attacker’s point of view. This is, in large part, due to the feature being available on all devices and supported by all apps since UI elements have the isImportantForAccessibility flag set to auto by default [19].

Furthermore, recent malware [10] has shown how to fool Google’s on-device scanner, Play Protect, through the use of accessibility as an attack vector. Since Play Protect was unable to detect the malicious code, this was therefore white-listed as a trusted application on the Play Store.

The use of overlays [20] has been proposed to increase attack stealthiness by reducing the visibility of any accessibility attack. However, this work primarily focused on showing how overlays can be used to trick users into enabling accessibility and hide any nefarious actions after that. That being said, recent malware has shown that less sophisticated methods, such as using deceit in their user prompts can suffice.

### Living Off the Land (LotL)

Campbell et al. [13] first introduced and coined the concept of LotL, even though several previous attacks already made use of this approach. Through their work, they concluded that “using a few built-in tools and a healthy dose of Powershell, an attacker can greatly reduce their forensic footprint”. The underpinning principle of LotL remains that of using white-listed system components through which to attain the malign attack objectives, as opposed to introducing malware components on the target system. To demonstrate the approach, they showed how classical attacks such as keylogging, C2, and privilege escalation are carried out on Windows systems. They then went on to compare these attack implementations to what they referred to as a minimalist approach, whereby only using built-in system tools they managed to obtain the same malicious outcomes while reducing their forensic footprint. In some cases they managed to obtain a fileless posture whereby no malicious executables are placed on the file system.

Due to the nature of its target device install-base, Android lacks similar administrative tools to allow an analogous tactic to work. However, Android’s accessibility services seem like the ideal substitute to implement a comparable strategy of providing access to all white-listed logic triggered via GUI actions. Even though such malware has to be delivered to the device in the form of an APK, subsequent attacks in this paper show that the APK may only be required during the initial stages of the attack, whit the following stages only requiring the white-listed apps.

### Elaborate Cybercrime Schemes

Malware is not the only tool available to cybercriminals. There are more elaborate schemes involving some other sort of deceit or impersonation. One example made infamous in recent years due to its role in many attacks involving the looting of cryptocurrency funds is that of SIM-swapping [21]. A SIM swap comprises an attacker successfully convincing a mobile operator to switch the victim’s phone number over to a different SIM card, one they own. By diverting the victim’s incoming messages, scammers can easily complete the 2FA checks commonly needed for moving cryptocurrencies out of exchanges. While the implications of these attacks can be significant, they tend to leave a more extensive forensic footprint across different sources. For example, in the case of SIM-swapping forensic analysts can now also rely on evidence left at the mobile operator, from simple audit entries down to CCTV footage from the mobile operator outlet where the SIM-swapping request occurred.

### Related Work

Obfuscation and anti-analysis [6] are popular approaches for achieving stealthiness while eluding detection at a malware sandbox level. However, detection and response tools can make use of deobfuscation [22] and other anti-anti-analysis tools and techniques to counter them. Yet some obfuscation techniques go to the extent of corrupting runtime structures to change the low-level semantics of the code [23], e.g. switch the behaviour of the innocuous JSONObject with that of LocationManager. While potentially bypassing app store and on-device scanning, access to the device location services, in this case, would ultimately get uncovered by any system API monitor. On the other hand, what would remain below the radar, even at this level of observation, is if malware delegated all of its location services misdemeanours to a benign app through accessibility services access.

While LotL is a well known stealth-enhancing threat on traditional platforms like MS Windows (with ongoing efforts towards novel LotL detection techniques [24]), not much work has focused on the possibility of LotL on Android. As a result, no LotL-specific analysis or forensic techniques have been studied or developed for Android. In this work, we are demonstrating that LotL can indeed be succesfully achieved on Android, through the abuse of accessibility services. Accessibility is a known threat on Android, and researchers are developing tools and techniques [19] aimed at mitigating some of these issues. However, the proposed measures are far from a complete solution to the problem and only tackle particular issues. Google developers have even tried to mitigate the threat by placing more stringent controls on the app store concerning accessibility apps. However, this decision had to be reverted [25] due to a backlash from the developer community.

Given the nature of accessibility, accessibility-enabled attacks can be found across platforms [26]. However, when compared to the second most popular mobile OS (iOS), Android attacks are much more prevalent due to the OS’s accessibility design. As opposed to granting accessibility to any app requesting the relevant permission, iOS takes a significantly more conservative approach with its UIAutomation framework. This feature set is part of the private API set, made available only to pre-packaged apps, and therefore its use is forbidden to third-party apps uploaded to the Apple Store.

## Accessibility Misuse

In this section, we will explain the general steps involved in a standalone accessibility attack, as well as the steps required in a comprehensive accessibility attack scenario which consists of several standalone accessibility attacks. We will then explain the different use cases of SMS abuse, C2-equipped malware, and crypto exchange theft, comparing implementations of a classical, non-accessibility implementation to different accessibility-enabled implementations.

### Threat Model

The relevant attacks for our work deal with hiding post-infection activity from detection and response tools. We assume that a malicious accessibility app managed to make its way to a user’s device undetected, and was subsequently granted the necessary permissions by the end-user. Surveys about malware in the wild have shown, time and again, that this scenario occurs way more frequently than what one would hope [27]. In the following sub-sections we first describe the attack steps common to any accessibility attacks aiming at transforming existing attacks into stealthier ones, we then present the specific use-cases of SMS abuse, C2-equipped malware, and a crypto exchange theft.

### Setting up Malicious Accessibility Services

Having a malicious accessibility service delegate functionality to a target victim app, or else attacking it directly in a malware-only setting, proceeds as follows. First, the app hosting the malicious accessibility service launches the victim app. This can be done using an Intent object, which is a predominantly asynchronous messaging component Android developers can use to request an action from another app component. Next, the accessibility service idles, waiting for the victim app to appear in the foreground. This event can be picked up by the appropriate filter being set inside the overridden onAccessibilityEvent() handler, at which point it can gain access to the relevant UI elements, e.g. buttons or edit texts. Subsequently, the accessibility service proceeds to traverse the tree of AccessibilityNodeInfo elements, calling performAction() among multiple other ways, to interact with the victim application.

For example, to exploit this flow for SMS abuse, the accessibility malware can first launch the default SMS app, passing the destination number and message text inside the Intent parameters. Next, the accessibility service starts monitoring the foreground activity, waiting for the SMS app to be in the foreground. Once it is, the accessibility service looks for the “send” button. It automatically completes the task by “clicking” on it through a performAction() call, without having to bring it to the user’s attention.

**Fig. 2.**
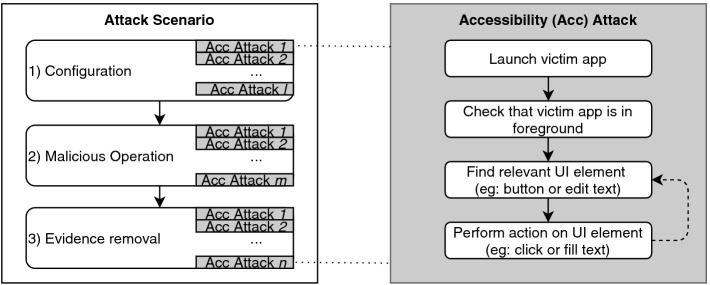
Accessibility attack scenario stages.

A comprehensive accessibility attack scenario ultimately comprises an entire sequence of such mimicked user actions, and with three main stages (see Fig. 2), where each one encompasses one or more accessibility attacks. The first one is the *Configuration* stage where the malware, once having tricked the user into granting the accessibility permission, proceeds to download the victim app, followed by any required user registration and after disabling notifications for additional stealth. All these steps can be carried out through accessibility services, and of course, are only required if the app is not already available on the device. The next *Malicious Operation* stage is victim-app-specific. It performs the interaction mentioned above steps, multiple times, to leverage whatever app functionality appeals to the attacker. The final *Evidence Removal* stage can optionally delete all remnants belonging to the attack steps, for example deleting any compromising sent SMS text message from its corresponding chat. However, it may be the case that the nature of the attack requires that the sent text message should be left on the device in order to confuse incident response.

It is noteworthy that the final *Evidence Removal* stage may even proceed to remove the malware app itself even before the attack is over. This could be the case where the victim app is one that provides some form of remote control, or else some kind of scheduled task, over the infected device. We refer to this scenario as *Full LotL*, meaning that the attacker manages to attain a malware-less posture on the device during an ongoing attack. In the upcoming use cases, we demonstrate two examples that abuse SMSonPC apps such as the popular Pushbullet app and remote administration apps such as the Teamviewer app for a similar purpose.

Even though the underlying accessibility service initiating the attack is running in the background, the accessibility attacks often force the victim app to run in the foreground. This can result in the victim seeing what is going on. However, through the use of overlay and speed tactics, we were able to reduce the impact of foreground activity further. The use of overlays to hide malicious activity has been proven to be very effective in hiding accessibility attacks [20].

### SMS Abuse Attack

*Non-accessibility Version.* On Android, two approaches allow apps to send SMSs. The first involves requesting the SEND_SMS permission and calling sendTextMessage() through SmsManager. The second involves using the default SMS app and passing an intent with the appropriate message parameters. The former is typically the preferred method since the latter requires user confirmation upon every SMS sending. It is worth noting that, in more recent Android versions, an SMS sending app is not allowed to delete an SMS unless the user explicitly configures the app as the default SMS application.

*LotL Version.* Through accessibility, a LotL version of the same attack becomes possible. The LotL SMS scenario in Table 1 describes the setup for each of the accessibility attack scenario stages, with the attacker sending text messages over SMS through the default messaging app. As opposed to the non-accessibility approach, this version does not require SEND_SMS permission or any of the associated API methods.

**Table 1.** Accessibility attack scenarios († = Accessibility attack)

Scenario	Configuration	Malicious operation	Evidence removal
LotL SMS	Targets default SMS app No config required	Send SMS$^{\dagger }$	Delete SMS$^{\dagger }$
Full LotL SMS	Install SMSonPC (Pushbullet)$^{\dagger }$ Enable Permissions$^{\dagger }$ Disable Notifications$^{\dagger }$ Login SMSonPC$^{\dagger }$	Send SMS through SMSonPC	Remove trojan$^{\dagger }$ OR Delete SMS$^{\dagger }$
LotL C2	Targets default web-browser No config required	Navigate to white-listed website$^{\dagger }$	Delete history$^{\dagger }$
Full LotL C2	Install Remote Admin app (Teamviewer)$^{\dagger }$ Enable permissions$^{\dagger }$ Disable notifications$^{\dagger }$ Login to Teamviewer$^{\dagger }$	Exfiltrate files from storage through Teamviewer remote control functionality	Remove trojan$^{\dagger }$
Crypto Exchange Theft	Assumes that the victim apps are already installed No configuration is required	Obtain 2FA token from 2FA app$^{\dagger }$ Perform withdrawal (passing 2FA as params)$^{\dagger }$	Remove trojan$^{\dagger }$

*Full LotL Version.* The Full LotL SMS scenario in Table 1 describes an alternative SMS abuse attack scenario setup, with added stealth. This time a SMSonPC app, e.g. Pushbullet, replaces the default messaging app. Once Pushbullet is smuggled onto the device, the attacker obtains full control over the SMS functionality on the victim device, and the malware is no longer required and thus deleted. We use the term Full LotL to refer to the fact that at this point, the attacker makes exclusive use of a benign app to successfully pull off the attack, without the need for malware persistence.

### C2-Equipped Malware

*Non-accessibility C2.* Widely deployed banking trojans typically come equipped with a C2 channel, providing attackers remote control over infected devices, with Android malware being no exception [10, 11]. While C2 channels can be used for all sorts of communication, exfiltration of stolen credentials as part of a phishing attack is a frequent feature in banking trojans. On the other hand, in Android, the shared storage location is used to store selfies, screenshots, and miscellaneous multimedia files. The READ_EXTERNAL_STORAGE permission is required for such operations. Transferring all stolen data back to the C2 server requires a custom network protocol or, preferably leveraging widely-used cloud messaging infrastructures, e.g. Firebase Cloud Messaging. HTTP tends to be the application protocol of choice, due to its compatibility with firewall settings. Whatever the approach, malware needs to request the INTERNET permission.

*LotL C2.* The LotL C2 scenario in Table 1 describes one possible setup for the different accessibility attack scenario stages, in order to employ a stealthy LotL C2 channel. In this case, the attacker uses the default web browser to send and receive data from the victim’s device. An advantage of this approach is that it does not require any extra permissions requests since they are already granted to the browser. Furthermore, malware can circumvent even the strictest URL black-list by connecting to a white-listed website rather than connecting directly to the C2 server.

*Full LotL C2*. Setting up a stealthy C2 can even be pushed further, for example, not requiring any malware to be present on the device beyond the configuration stage. As shown in the Full LotL C2 scenario shown in Table 1, the accessibility malware can simply install a white-listed remote administration app to control the device and exfiltrate user data.

### Crypto Exchange Theft

*SIM Swapping.* A SIM swap is when someone convinces a mobile operator to switch the victim’s phone number over to a SIM card they own. By diverting the victim’s incoming messages, scammers can easily complete the text-based 2FA checks that typically protect crypto exchange accounts. In combination with stolen passwords from a phishing attack, this leaves the victim’s crypto wallet up for grabs.

*Malware-Only Attack.* 2FA-bypassing malware is the holy grail of banking trojans. An Android accessibility abuse makes this possible. The setup of this scenario is shown in the Crypto Exchange Theft scenario in Table 1. In this case, it is noteworthy that SMS 2FA tokens are gradually being replaced by more secure alternatives [28], with 2FA apps being the most popular choice. However, even these can be bypassed with accessibility attacks, in the same spirit. Perhaps only hardware tokens or biometric-based ones present an exception, although creative social engineering tricks can probably overcome these extra (and as yet uncommon) security measures.

**Fig. 3.**
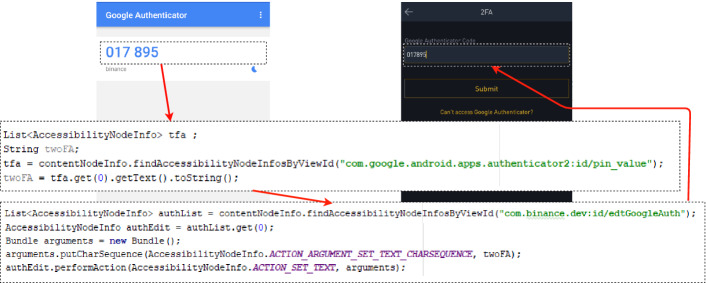
2FA token theft and use.

In this scenario, the accessibility trojan obtains the 2FA token from the 2FA app or SMS and then passes the stolen token as a parameter in the withdrawal process. Fig. 3 shows how after using an intent to launch the 2FA app (Google Authenticator), the trojan reads the text value of the token by finding the text value of the view com.google.android.apps.authenticator2:id/pin_value using the accessibility command findAccessibilityNodeInfosByViewId. After obtaining the 2FA token, the malware then opens the victim’s exchange app and clicks its way through the withdrawal process. Once the 2FA entry is prompted, the trojan then passes the stolen value in the appropriate edit text.

## Forensic Footprint Comparisons

Here we demonstrate the reduced forensic footprint in all of our attack examples, by comparing the artefacts left behind in each case. The forensic footprint is compared across several analysis probes. Unless noted otherwise, all sources of evidence are supplied by MobSF probes. All attacks were executed on actual Android devices (Nexus 5x, Samsung Galaxy S8, and Samsung A30s) as well as on Android emulators ranging from Android API levels 23 to 29. The artefacts taken into consideration are as follows: APK-derived requested Permissions (P), and API and Library classes/methods (ALc); Sandbox execution-derived API and Library method calls invoked (ALe), Network traffic (N), non-Volatile Memory (nVM) data, Logcat (L) and Dumpsys (D) entries.

We ranked the outcome of the forensic footprint for each of the artefacts between 0–2. A 0 label indicates that the malware does not leave any forensic footprint behind. In contrast, a value of 2 indicates that there is enough forensic footprint to attribute the malicious behaviour to the malware through clear evidence. A value of 1 indicates that a forensic footprint exists, however it does not suffice to attribute the malicious behaviour to the malware. In the cases of probes P and ALc, attribution is determined through the binary on which the analysis is occurring. Whereas the remaining probes depend on process IDs for attribution. Additionally, N also makes use of process ID and src/dest port correlation, and nVM makes use of the app’s local storage.

All attacks from Sect. 3 were implemented in the Metasploit pentest framework, as part of the Android Meterpreter payload. The post-exploitation commands used were send_sms for SMS sending and commands from the stdapi command set such as download for C2 data exfiltration. The accessibility attacks required an alternative implementation for each of them, specifically targeting: Pushbullet (package: com.pushbullet.android, ver:18.2.35) and Google Messages (package: com.google.android.apps.messaging, ver:5.7.097) in the case of SMS sending, and Teamviewer Host (package: com.teamviewer.host.market, ver: 15.6.51) and Google Chrome (package: com.android.chrome, ver:81.0.4044.117) in the case of C2. Finally, the Crypto exchange theft was tested on Binance (package: com.binance.dev, ver:1.21.1).

### Results

**Fig. 4.**
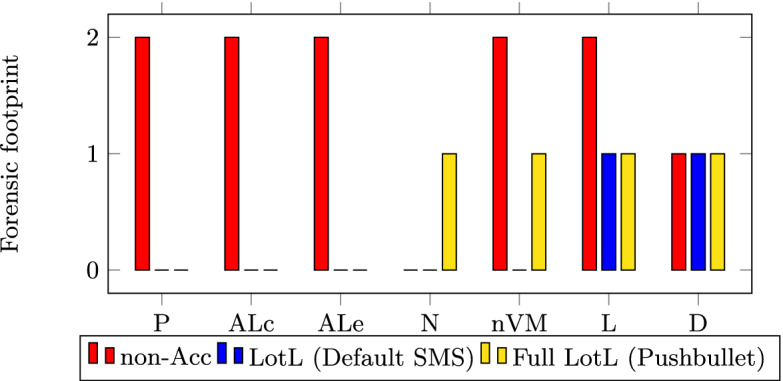
Forensic footprints - SMS abuse attacks.

*SMS Abuse.* Figure 4 shows the SMS abuse forensic footprint comparison results. It reveals LotL’s reduced forensic footprint across the various probes. One instance where the LotL approach leaves more forensic evidence than the non-accessibility approach is the network activity during the Pushbullet attack. This observation is due to Pushbullet having first to receive a network command before it can send text messages. That said, the network activity is not attributed to the malicious accessibility app, but rather to Pushbullet. In fact, in this Full LotL attack version, the accessibility malware is not even on the device anymore at the point when the text messages are sent.

**Fig. 5.**
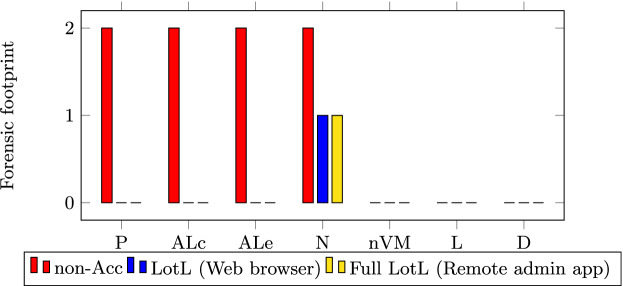
Forensic footprints - C2-equipped malware attacks.

*C2-Equipped Malware.* Figure 5 shows the C2 forensic footprint comparison, clearly displaying LotL’s reduced forensic footprint across the various probes.

*Crypto Exchange Theft.* Had a SIM-swapping approach been followed for pulling off this attack, the expected result is one where the attacker ample leaves forensic evidence at the mobile provider, possibly including CCTV footage of the person buying the SIM. Moreover, at the exchange site, new logs originating from the attacker’s IP rather than the victim’s could also be observed. That is one of the reasons why Binance triggers an alarm every time an authentication request comes from new/unknown IP addresses. Once IP data is obtained, forensic analysts can even dig deeper and request service provider logs to identify the source of the crime. Cybercriminals can make use of tools like anonymous proxies or VPNs in order to cover their tracks, however, an alarm will still be triggered for every new/unknown IP used.

**Fig. 6.**
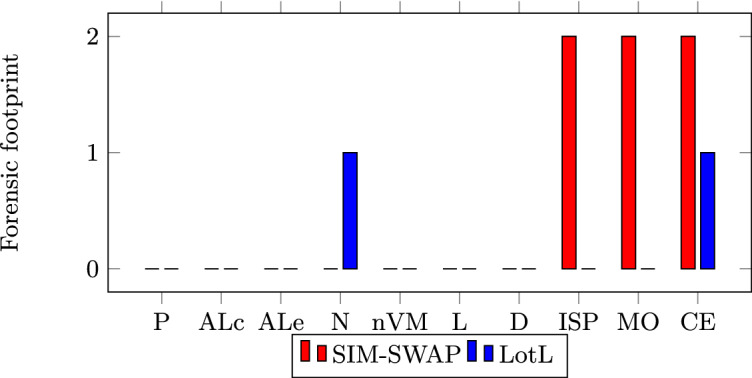
Forensic footprints - Crypto exchange theft.

On the other hand, the malware-only version of the exchange withdrawal attack has the advantage that it only leaves forensic evidence on the device, and even in this case, the forensic evidence on the device is already significantly reduced. The only left artefact is network activity between the victim device and the exchange. However, since the victim would have already been using the app, this would seem like perfectly normal network activity. Proving in a forensically sound manner that it was not the user who executed the withdrawal could be quite tricky, especially if the accessibility trojan is removed afterwards. From the ISP, crypto exchange, and mobile operator perspective, all of the forensic artefacts point towards legitimate activity from the victim’s device. This could be very problematic when dealing with cases of financial fraud. Figure 6 shows the forensic footprint comparison results. In this case, we also consider external sources, namely: ISP, crypto exchange (CE), and mobile operator (MO).

We have responsibly disclosed the possibility of these attacks to two of the most popular crypto exchanges, and both acknowledged the issue. To their credit, after our communication and only in a matter of days Binance took appropriate measures by disabling the accessibility feature for critical UI elements involved in the withdrawal process. We tested the attack against the updated version of their application and confirmed that it was able to block our initial accessibility withdrawal attack. The other exchange awarded us a bug bounty on the Hackerone platform [29] and seems to be in the process of mitigating the issue. It is important to note that this is not necessarily a bug or vulnerability from the part of the exchanges, but rather an OS feature that can be easily misused for stealing funds if no new countermeasures are implemented.

## Discussion and Conclusion

Given the potential for stealthiness in accessibility attacks, their forensic analysis requires not only leveraging the state-of-the-art in mobile forensics but also extending it further concerning ephemeral evidence inside volatile memory. Stealthy malware is unlikely to leave any traces on disk. However, it cannot avoid leaving marks on volatile memory at one point or another. The likely brief presence in memory of relevant artefacts, which could be indicators of compromise, call for a just-in-time collection approach [30]. The challenges with this strategy are, nevertheless, many. The memory collection has to be done in a timely manner, and overheads have to be kept minimal while still being able to locate and parse the required evidence. Additionally, the method employed must ensure that it does not cause the app to crash nor allow the malware to detect that the analysis is taking place. In terms of prevention, hardened authentication mechanisms such as physical 2FA tokens and interactive CAPTCHAs can help reduce the risk of malicious apps performing sensitive operations on the user’s behalf.

Accessibility apps have become a “double-edged sword”, as they bring significant security threats to users. Malicious accessibility apps are able to perform sensitive operations by hijacking trusted white-listed apps easily, and more importantly, they can elude detection by leaving behind a significantly reduced forensic footprint. In this paper, we first studied the security risks of accessibility apps employing LotL tactics, and those that offer a malware-only substitute for attacks typically requiring more elaborate schemes. To demonstrate the reduced forensic footprint, we compared the analysis of accessibility malware with non-accessibility variants. The experimental results showed that across several analysis probes, accessibility malware could significantly reduce the forensic footprint when performing malign operations. Our work exposes the severe security threats and outlines the forensic implications of accessibility trojans. We have notably responsibly disclosed the impact of our research on two major cryptocurrency exchanges. Our work has led to one (Binance) immediately upgrading the security of its mobile app while the other is currently working on it. Given the severity of the threats posed, as well as the powerful stealth capabilities of accessibility attacks, Google may need to reconsider the openness of its accessibility services in the short term, before cybercriminals start exploiting them widely.
